# Supplementary material for: Phenome-wide Mendelian randomisation analysis of 378,142 cases reveals risk factors for eight common cancers
Source: Nat Commun. 2024 Mar 25;15:2637. doi: 10.1038/s41467-024-46927-z (PMC10963765; doi:10.1038/s41467-024-46927-z)
Supplement: Supplementary file 1 — Supplementary Information [file 41467_2024_46927_MOESM1_ESM.pdf]

Risk factors for eight common cancers revealed from a phenome-wide Mendelian randomisation analysis of 378,142 cases

Went, Sud, Mills, Hyde *et al*

Supplementary Figures

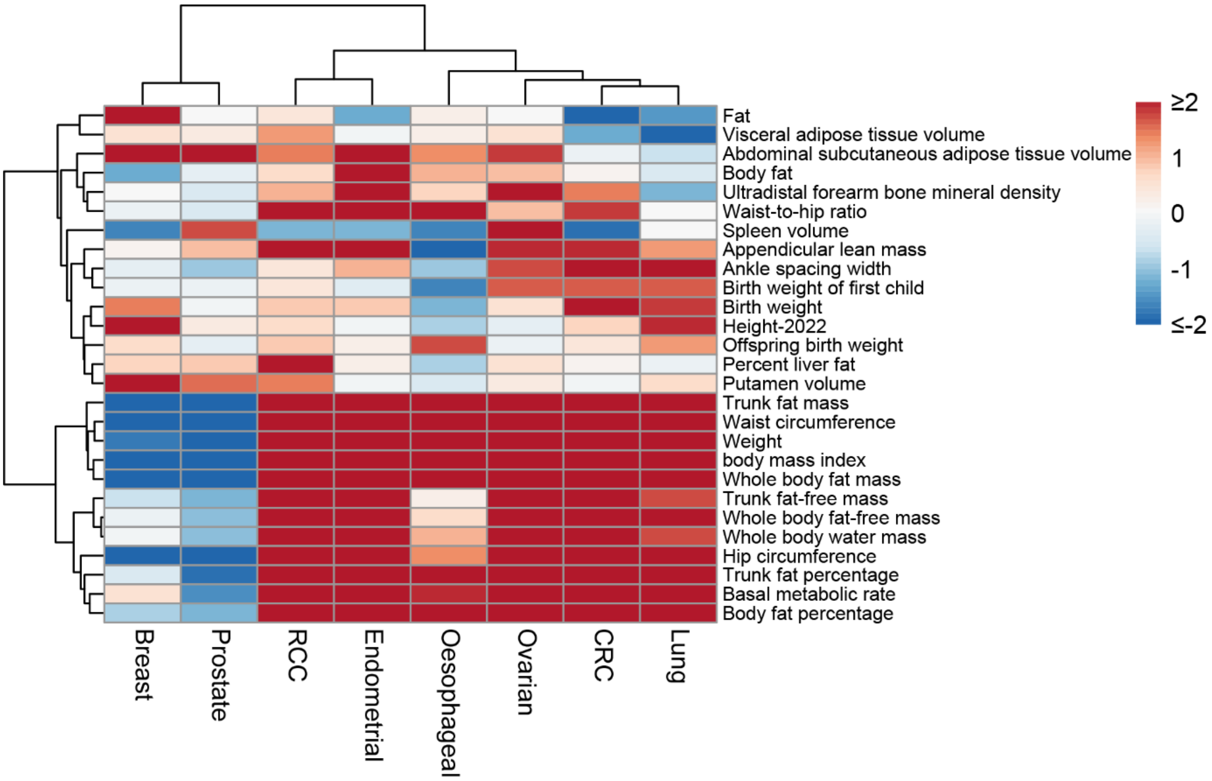

Supplementary Figure 1: Heatmap and dendrograms showing clustering of causal associations between anthropometric traits and cancer risk. Heatmap based on Z-statistics using the row and column clustering implemented in the pheatmap function within R. Colours correspond to the strength of associations and their direction (red positive association with risk, blue inverse association with risk). Only traits showing an association for at least one cancer type are shown.

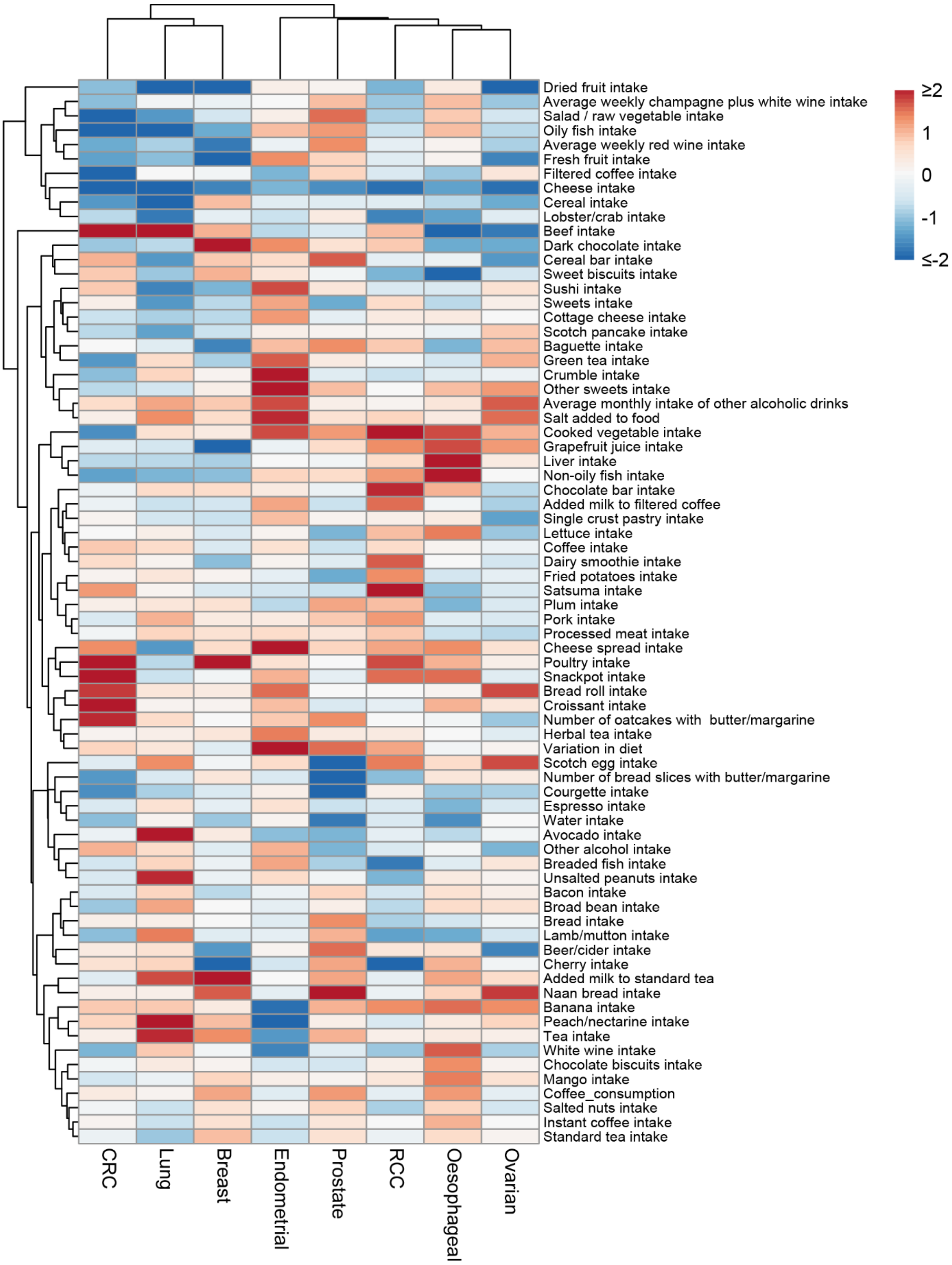

Supplementary Figure 2: Heatmap and dendrograms showing clustering of causal associations between dietary intake traits and cancer risk. Heatmap based on Z-statistics using the row and column clustering implemented in the pheatmap function within R. Colours correspond to the strength of associations and their direction (red positive association with risk, blue inverse association with risk). Only traits showing an association for at least one cancer type are shown.

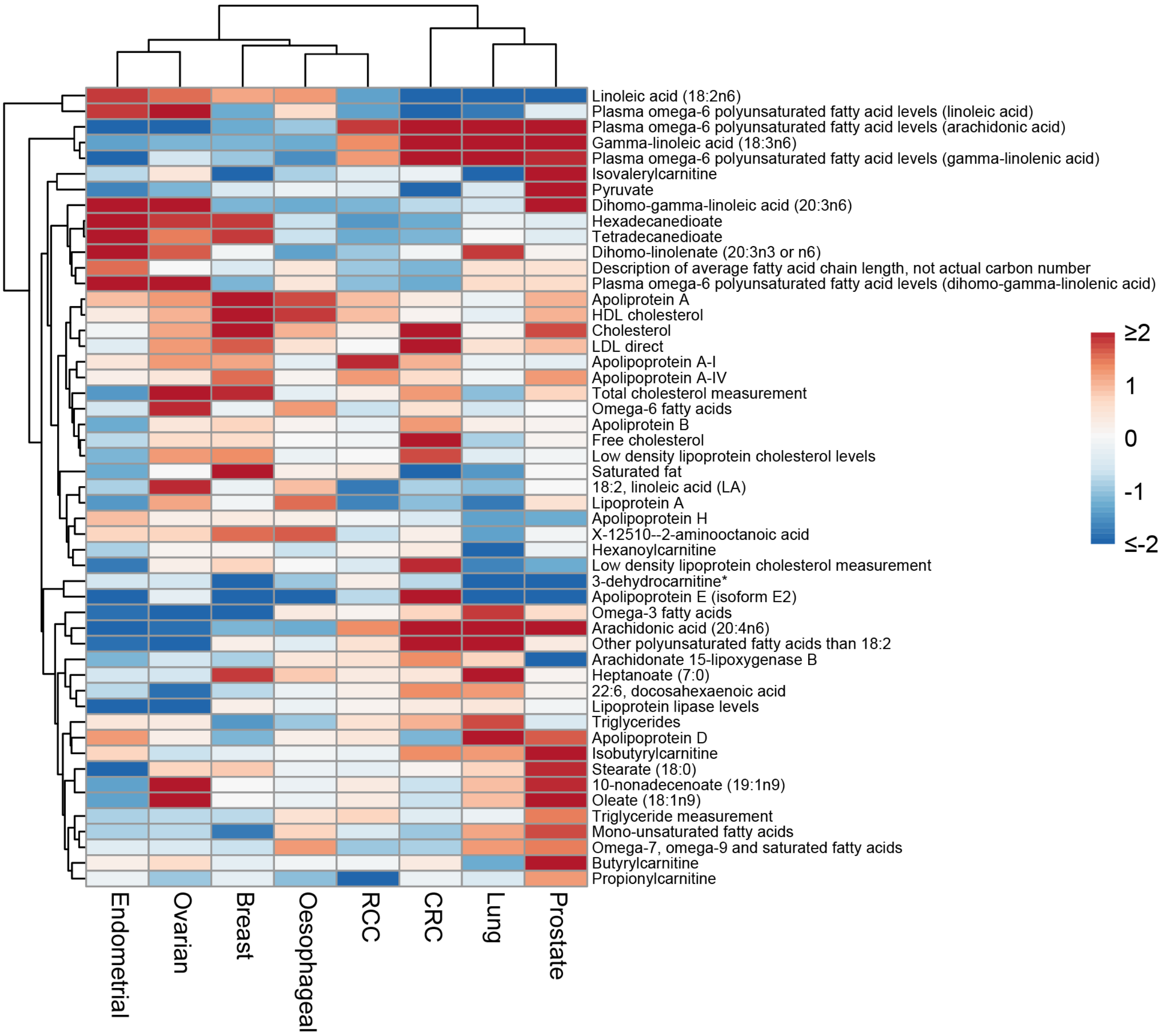

Supplementary Figure 3: Heatmap and dendrograms showing clustering of causal associations between fatty acids and cancer risk. Heatmap based on Z-statistics using the row and column clustering implemented in the pheatmap function within R. Colours correspond to the strength of associations and their direction (red positive association with risk, blue inverse association with risk). Only traits showing an association for at least one cancer type are shown.

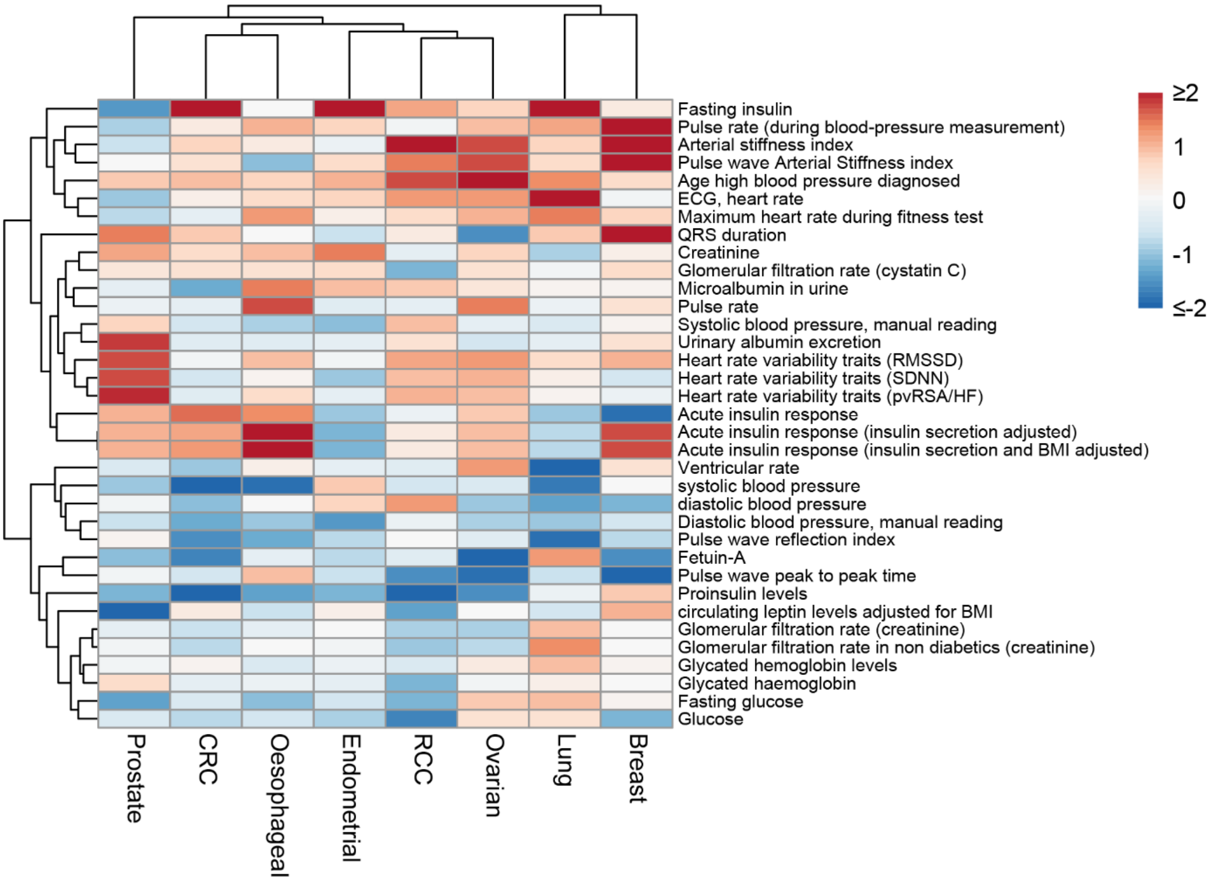

Supplementary Figure 4: Heatmap and dendrograms showing clustering of causal associations between cardiometabolic traits and cancer risk. Heatmap based on Z-statistics using the row and column clustering implemented in the pheatmap function within R. Colours correspond to the strength of associations and their direction (red positive association with risk, blue inverse association with risk). Only traits showing an association for at least one cancer type are shown.

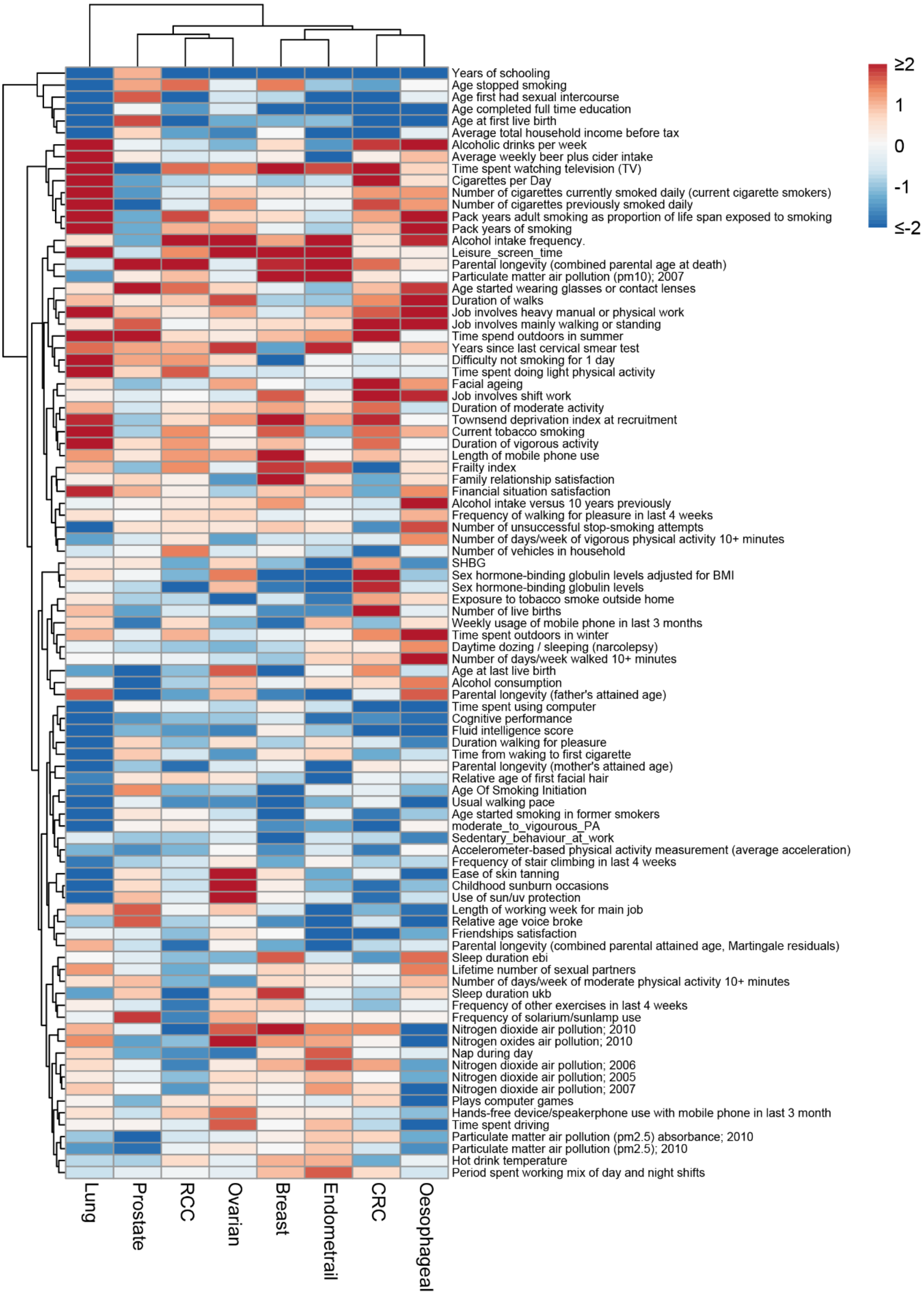

Supplementary Figure 5: Heatmap and dendrograms showing clustering of causal associations between lifestyle traits and cancer risk. Heatmap based on Z-statistics using the row and column clustering implemented in the pheatmap function within R. Colours correspond to the strength of associations and their direction (red positive association with

risk, blue inverse association with risk). Only traits showing an association for at least one cancer type are shown.

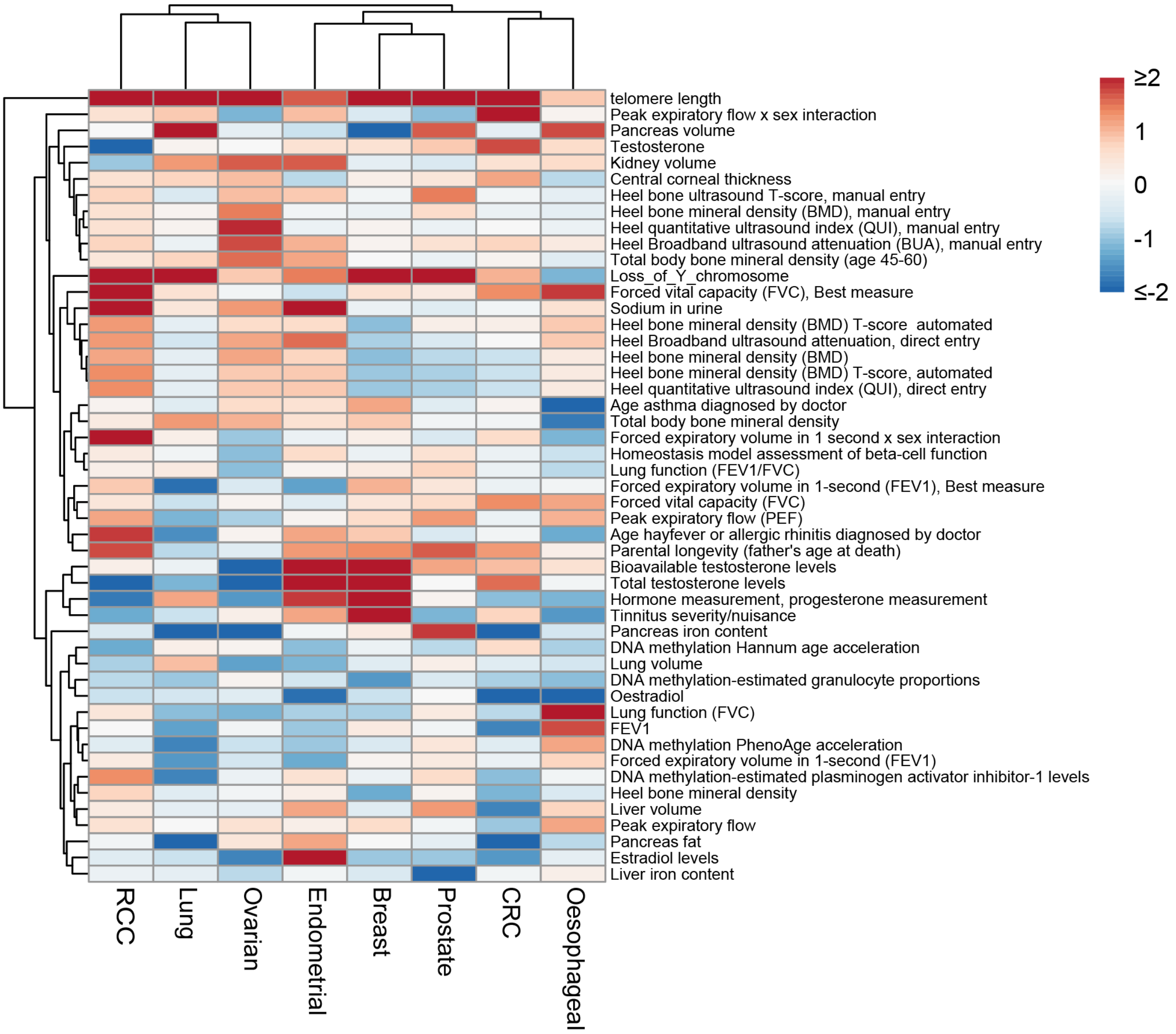

Supplementary Figure 6: Heatmap and dendrograms showing clustering of causal associations between miscellaneous traits and cancer risk. Heatmap based on Z-statistics using the row and column clustering implemented in the pheatmap function within R. Colours correspond to the strength of associations and their direction (red positive association with risk, blue inverse association with risk). Only traits showing an association for at least one cancer type are shown.

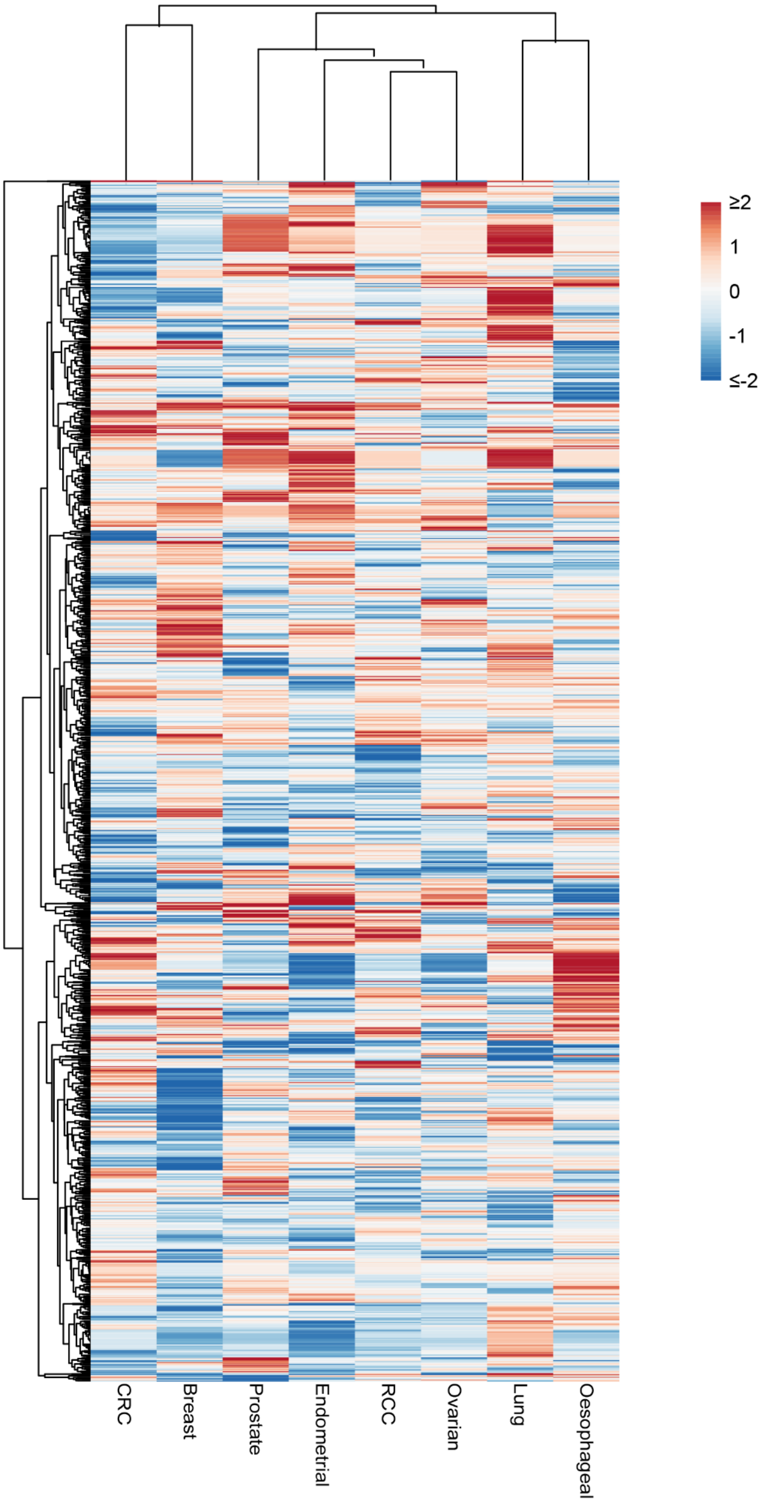

Supplementary Figure 7: Heatmap and dendrograms showing clustering of causal associations between proteomic and metabolomic traits and cancer risk. Heatmap based on Z-statistics using the row and column clustering implemented in the pheatmap function within R. Colours correspond to the strength of associations and their direction (red positive association with risk, blue inverse association with risk). Only traits showing an association for at least one cancer type are shown. Trait names are removed due to the large number of traits in the figure.

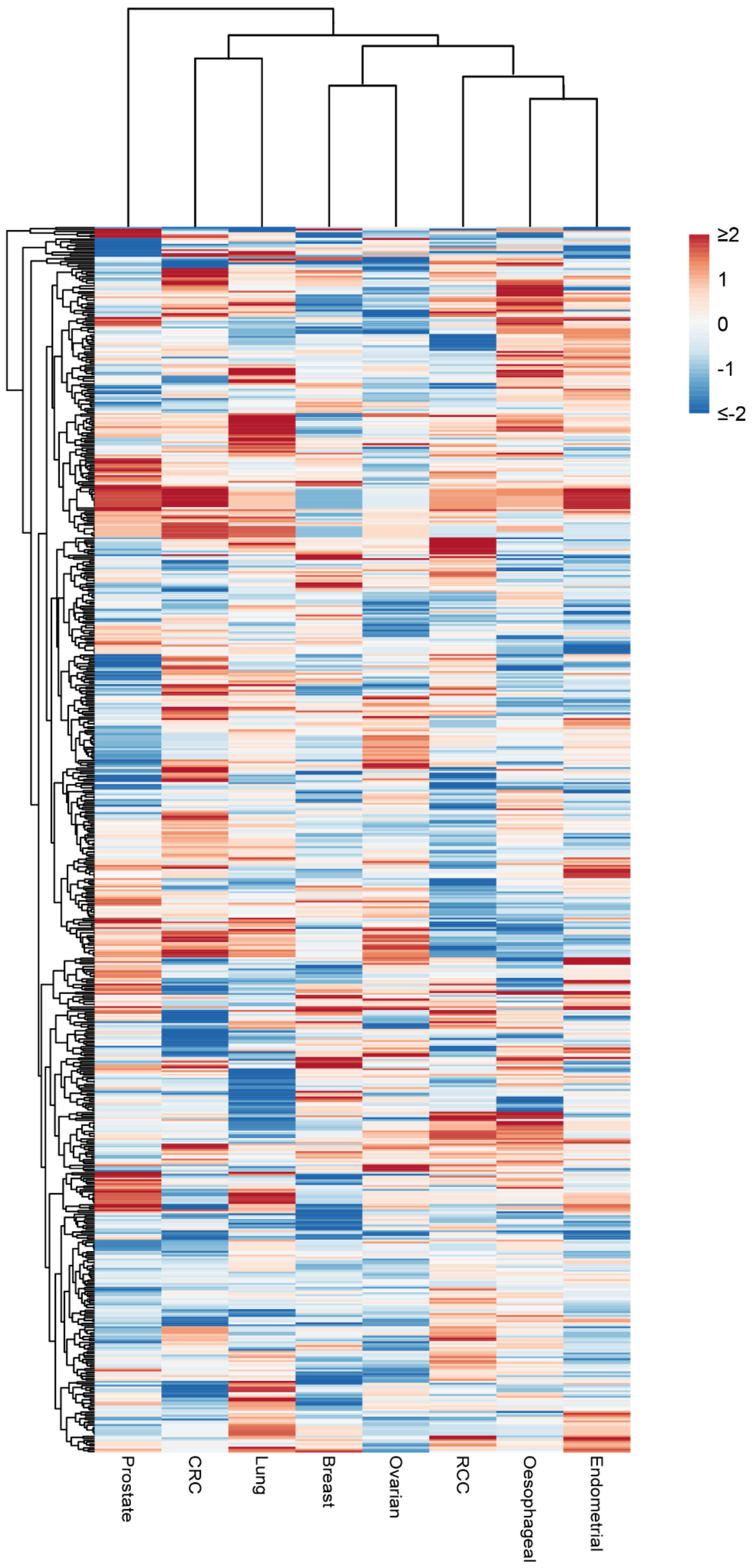
≥2
1
0
-1
≤-2
Prostate
CRC
Lung
Breast
Ovarian
RCC
Oesophageal
Endometrial

Supplementary Figure 8: Heatmap and dendrograms showing clustering of causal associations between immune and inflammatory traits and cancer risk. Heatmap based on Z-statistics using the row and column clustering implemented in the pheatmap function within R. Colours correspond to the strength of associations and their direction (red positive association with risk, blue inverse association with risk). Only traits showing an association for at least one cancer type are shown. Trait names are removed due to the large number of traits in the figure.
