# Supplementary material for: Phenome-wide Mendelian randomisation analysis of 378,142 cases reveals risk factors for eight common cancers
Source: Nat Commun. 2024 Mar 25;15:2637. doi: 10.1038/s41467-024-46927-z (PMC10963765; doi:10.1038/s41467-024-46927-z)
Supplement: Supplementary file 3 — Description of Additional Supplementary Files [file 41467_2024_46927_MOESM3_ESM.pdf]

Description of Additional Supplementary Files

Supplementary Data 1

Description: Details of filtering applied to instrumental variables used in the Mendelian randomisation analysis. Final row (after all filtering steps) indicates number of traits analysed for each cancer type.

Supplementary Data 2

Description: Traits considered in MR analysis. Power for each trait calculated across a range of odds ratios. SNP, single nucleotide polymorphism; ORSD, odds ratio per standard deviation unit increase in the putative risk factor; N, trait sample size; R2, estimate of proportion of variance explained; PMID, Pubmed ID.

Supplementary Data 3

Description: Causal estimates from the Mendelian randomisation analysis for each continuous trait and cancer risk. Results reported as odds ratios (ORSD) and 95% confidence intervals (CIs) per genetically predicted standard deviation (SD) unit increase in the risk factor. Two-sided P-value reported. Wald ratio reported if only 1 SNP available for use in MR analysis. Inverse-variance weighted random effects model only computed if ≥2 SNPs available for use in MR analysis. SNP, single nucelotide polymorphism; LCI, lower 95% confidence interval; UCI, upper 95% confidence interval; N, trait sample size.

Supplementary Data 4

Description: Leave one out inverse variance weighted random-effects MR analysis for each exposure trait and cancer type. Results reported as effect size beta and standard error (SE) per genetically predicted standard deviation (SD) unit increase in the risk factor. Two-sided P-value reported. Leave-one-out analysis not conducted if ≤2 SNPs available for use in MR analysis. N, trait sample size; MR, Mendelian randomisation. Traits with NA indicate those with too few SNPs to perform leave-one-out analysis,

Supplementary Data 5

Description: Single SNP analysis for fatty acid traits and their effect on colorectal cancer risk. Wald ratio results for each fatty acid SNP reported as effect size (beta) and standard error (SE) per genetically predicted standard deviation (SD) unit increase in the risk factor. Two-sided P-value calculated.

Supplementary Table 6

Description: Results of Bayesian colocalisation between exposure and outcome. Results for colocalisation analysis for proteomic traits which had an IV located cis (+/- 1Mb) of the gene target using coloc. This enumerates the four possible configurations of causal variants for two traits, calculating support for each model based on a Bayes factor. Prior probabilities of p1, p2 = 1e-04 and p12 = 1e-05 were used and a posterior probability ≥0.80 was considered as supporting a specific model. H0: No association with either trait, H1: Association with trait 1, not with trait 2, H2: Association with trait 2, not with trait 1, H3: Association with trait 1 and trait 2, two independent SNPs, H4: Association with trait 1 and trait 2, one shared SNP.

Supplementary Data 7

Description: Causal estimates for each Mendelian randomisation method for each binary trait and cancer risk. Results reported as effect size (beta) and standard error (SE) per genetically predicted standard deviation (SD) unit increase in the risk factor. Two-sided P-value shown. Wald ratio reported if only 1 SNP available for use in MR analysis. Inverse-variance weighted random effects model only computed if ≥2 SNPs available for use in MR analysis. SNP, single nucleotide polymorphism; N, trait sample size.

Supplementary Data 8

Description: Results of the multivariable Mendelian randomisation (MVMR) analysis. We used the mv_multiple function in the TwoSampleMR package to perform multivariable MR (MVMR), which was applied to investigate which biologically related traits had independent pleiotropic effects on a specific cancer. We restricted our MVMR analyses to traits which had ≥2 IVs and for which we had access to full summary statistics required for the analysis. Statistical analyses were performed using the TwoSampleMR package v0.5.6 (https://github.com/MRCIEU/TwoSampleMR). ORMR; odds ratios per genetically predicted SD unit increase in each trait for MR analysis, ORMVMR; odds ratios per genetically predicted SD unit increase in each putative risk factor for MVMR analysis.

* these are using the largest height dataset for which we don't have full data available. Breast Height 2022 ORMR= 1.1, PMR=3.86x10-3. CRC Height 2022 ORMR= 1.03, PMR=0.44

Supplementary Data 9

Description: Literature triples identified across eight different cancer types and Mendelian randomisation defined risk factors using SemMedDB.

Supplementary Data 10

Description: Stratification of literature space size by trait category.

Supplementary Data 11

Description: List of potential mediators for each trait identified using SemMedDB.

Supplementary Data 12

Description: Effect allele, effect allele frequency and effect sizes for association of SNPs used as instrumental variables. Harmonised summary data for instrumental variables used in MR analysis. The effect estimates (betas), standard errors (SE) and effect allele frequencies are from the publications indicated in the Pubmed ID column or in the Data Availability section. Beta values represent the per-allele effect of a SNP on a trait in standard deviation units of the trait. SNP, single nucleotide polymorphism; PMID, Pubmed ID; EA, effect allele; OA, other allele; EAF, effect allele frequency; SE, standard error; MR Mendelian randomisation.

Supplementary Data 13

Description: Causal estimates from the Mendelian randomisation analysis for each continuous trait and breast cancer subtype risk. Results reported as odds ratios (ORSD) and 95% confidence intervals (CIs) per genetically predicted standard deviation (SD) unit increase in the risk factor. Two-sided P-value reported. Wald ratio reported if only 1 SNP available for use in MR analysis. Inverse-variance weighted random effects model only computed if ≥2 SNPs available for use in MR analysis. SNP, single nucleotide polymorphism; LCI, lower 95% confidence interval; UCI, upper 95% confidence interval; N, trait sample size luminalBHer2neg, HER-2 negative Luminal B; tripleneg, triple negative; luminalA, luminal A; luminalB, luminal B; HER2enriched, HER-2 enriched.

Supplementary Data 14

Description: Causal estimates from the Mendelian randomisation analysis for each continuous trait and lung cancer subtype risk. Results reported as odds ratios (ORSD) and 95% confidence intervals (CIs) per genetically predicted standard deviation (SD) unit increase in the risk factor. Two-sided P-value reported. Wald ratio reported if only 1 SNP available for use in MR analysis. Inverse-variance weighted random effects model only computed if ≥2 SNPs available for use in MR analysis. SNP, single nucelotide polymorphism; LCI, lower 95% confidence interval; UCI, upper 95% confidence interval; N, trait sample size.

Supplementary Data 15

Description: Causal estimates from the Mendelian randomisation analysis for each continuous trait and ovarian cancer subtype risk. Results reported as odds ratios (ORSD) and 95% confidence intervals (CIs) per genetically predicted standard deviation (SD) unit increase in the risk factor. Two-sided P-value reported. Wald ratio reported if only 1 SNP available for use in MR analysis. Inverse-variance weighted random effects model only computed if ≥2 SNPs available for use in MR analysis. SNP, single nucleotide polymorphism; LCI, lower 95% confidence interval; UCI, upper 95% confidence interval; N, trait sample size.

Supplementary Data 16

Description: Weighted median estimate and mode-based estimates for each exposure trait and cancer type. Results reported as effect size (beta) and standard error (SE) per genetically predicted standard deviation (SD) unit increase in the risk factor. Two-sided P-values shown. Weighted median estimator and mode-based estimate only computed if ≥3 SNPs available for use in MR analysis. SNP, single nucleotide polymorphism; SE, standard error; N, trait sample size.

Supplementary Data 17

Description: MR-Egger regression analysis for each exposure trait and cancer type.

MR-Egger estimate only computed if ≥5 SNPs available for use in MR analysis. Shown are tests for two-sided P-values. SE, standard error; N, trait sample size; MR, Mendelian randomisation.

Supplementary Data 18

Description: MR Steiger analysis for each continuous exposure trait and cancer type.

Shown are two-sided tests for P-value. R2, proportion of variance explained; SNP, single nucleotide polymorphism; MR, Mendelian randomisation.

Supplementary Data 19

Description: Lifetime risk estimates for each cancer type used to estimate the proportion of variance explained.

Supplementary Data 20

Description: Conditional F-statistics for traits included in multivariable MR analysis. Conditional F-statistics for the traits were calculated using the condFstat function in the MendelianRandomzation package (https://cran.r-project.org/web/packages/MendelianRandomization/index.html).

Supplementary Data 21

Description: Genetic correlation estimates for traits included in multivariable MR analysis. Genetic correlation between traits was estimated using Linkage-Disequilibrium Adjusted Kinships (LDAK) software.

Supplementary Data 22

Description: The hierarchical levels of statistical significance used to classify associations in the MR analysis.† as indicated by MR Steiger test. IVW-RE, Inverse variance weighted- random effects test; WME, Weighted mode estimate; MBE, Weighted mode-based estimate.

Supplementary Data 23

Description: Causal estimates for each Mendelian randomisation method for each binary trait and breast cancer subtype risk. Results reported as effect size (beta) and standard error (SE) per genetically predicted standard deviation (SD) unit increase in the risk factor. Two-sided P-value shown. Wald ratio reported if only 1 SNP available for use in MR analysis. Inverse-variance weighted random effects model only computed if ≥2 SNPs available for use in MR analysis. SNP, single nucleotide polymorphism; N, trait sample size; luminalBHer2neg, HER-2 negative Luminal B; tripleneg, triple negative; luminalA, luminal A; luminalB, luminal B; HER2enriched, HER-2 enriched.

Supplementary Data 24

Description: Causal estimates for each Mendelian randomisation method for each binary trait and lung cancer subtype risk. Results reported as effect size (beta) and standard error (SE) per genetically predicted standard deviation (SD) unit increase in the risk factor. Two-sided P-value shown. Wald ratio reported if only 1 SNP available for use in MR analysis. Inverse-variance weighted random effects model only computed if ≥2 SNPs available for use in MR analysis. SNP, single nucleotide polymorphism; N, trait sample size.

Supplementary Data 25

Description: Causal estimates for each Mendelian randomisation method for each binary trait and ovarian cancer subtype risk. Results reported as effect size (beta) and standard error (SE) per genetically predicted standard deviation (SD) unit increase in the risk factor. Two-sided P-value shown. Wald ratio reported if only 1 SNP available for use in MR analysis. Inverse-variance weighted random effects model only computed if ≥2 SNPs available for use in MR analysis. SNP, single nucleotide polymorphism; N, trait sample size.
